# Supplementary figures and images for: Zebrafish genetic model of neuromuscular degeneration associated with Atrogin-1 expression
Source: PLoS Genet. 2026 Jan 9;22(1):e1012019. doi: 10.1371/journal.pgen.1012019 (PMC12810921; doi:10.1371/journal.pgen.1012019)

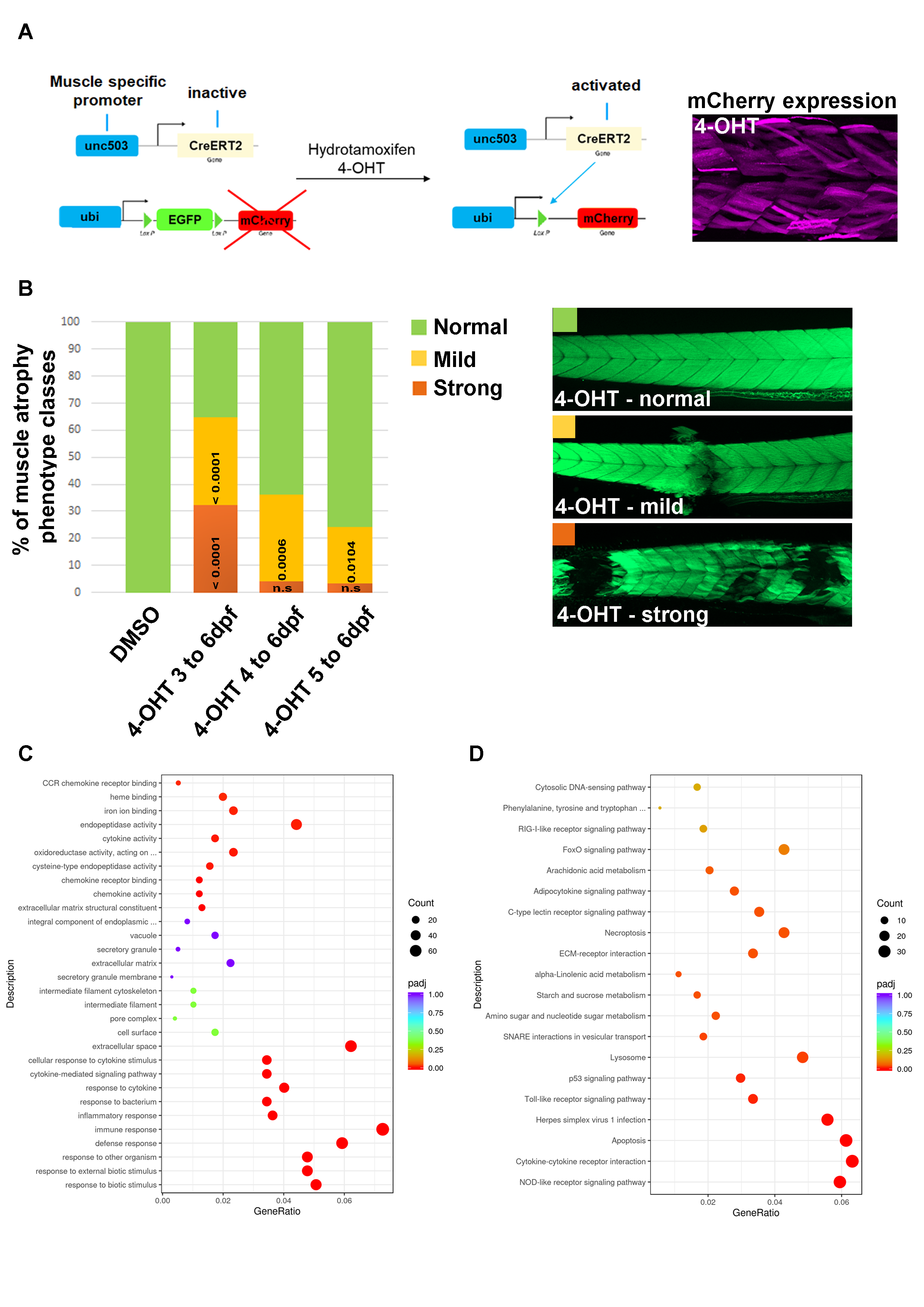

Supplement: S1 Fig — (A) Schematic representation of the method used to validate Tg(503unc:creERT2) transgenic line with the Tg(ubi:switch) transgene. Efficient recombination of the loxP sites following 4-OHT treatment leads to mCherry expression in muscle fibers. (B) Quantification of proportion of the different phenotypes, as measured via phalloidin incorporation, observed in atrofish larvae treated with DMSO (n = 29), and after 24 (n = 29), 48 (n = 28), or 72 (n = 34) hours of 4-OHT treatment. Significance is determined by contingency Fisher’s exact test. (C) GO (Gene Ontology) analysis between control atrofish larvae (DMSO-treated) and atrofish larvae treated with 4-OHT for 24 hours. (D) KEGG analysis between control atrofish larvae (DMSO-treated) and atrofish larvae treated with 4-OHT for 24 hours. Error bars represent s.d. (TIF) [file pgen.1012019.s001.tif]

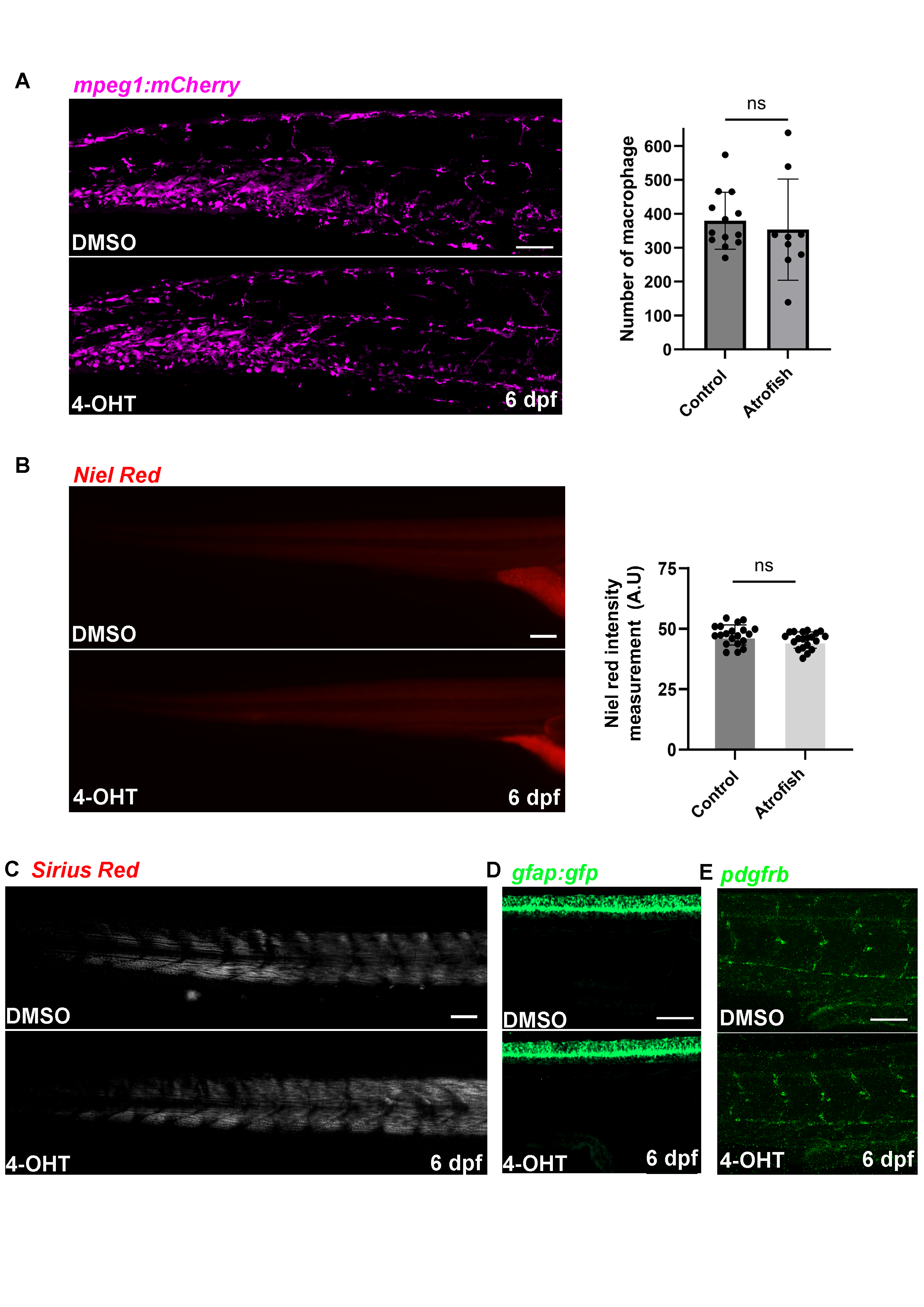

Supplement: S2 Fig — Confocal projections of DMSO-treated atrofish larvae (top) or atrofish larvae treated with 4-OHT for 48 hours (bottom) at 6 dpf. (A) The number of macrophages was quantified using the mpeg1:mCherry transgenic line (DMSO; n = 13 and 4-OHT; n = 10). (B) Fat deposition was analyzed using Nile red incorporation (DMSO; n = 20 and 4-OHT; n = 20). Statistical significance is determined by multiple t-test, two-tailed, unpaired. Error bars represent s.d. (C) Representative picture of Sirius red staining to analyze collagen deposition in skeletal muscle tissue. (D) Representative images of gfap:gfp expression. GFAP expression was analyzed using the gfap:gfp transgenic line. (E) Representative image of pdgfrb expression. pdgfrb expression was analyzed using HCR fluorescent in situ hybridization. Scale bars: 100 μm. (TIF) [file pgen.1012019.s002.tif]

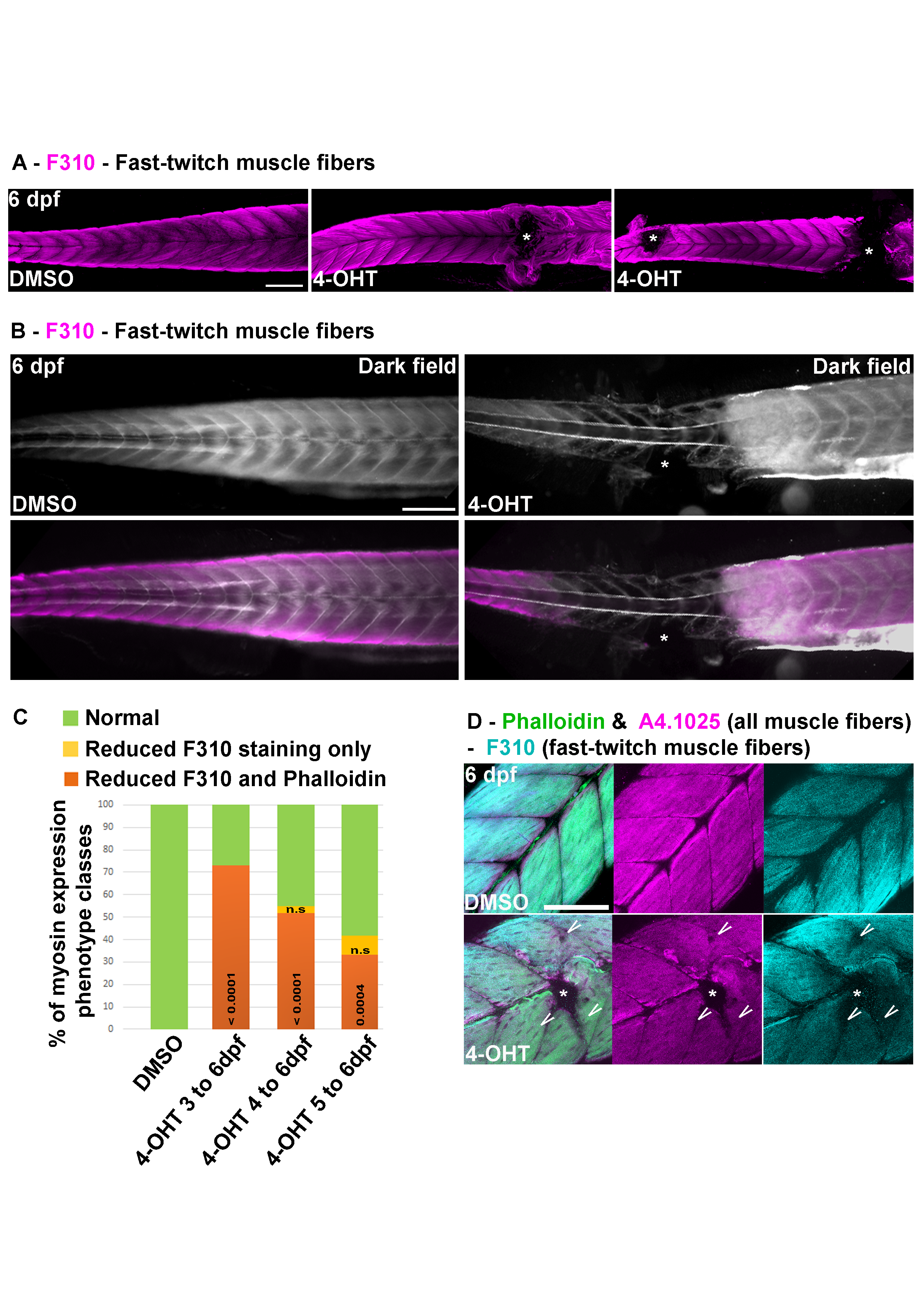

Supplement: S3 Fig — (A) Apotome sections of DMSO-treated atrofish larvae (left) or atrofish larvae treated with 4-OHT for 72 hours (right) at 6 dpf showing reduced F310 immunolabelling (purple) in trunk skeletal muscles. (B) Apotome sections of DMSO-treated atrofish larvae (left) or atrofish larvae treated with 4-OHT for 72 hours (right) at 6 dpf showing reduced F310 immunolabelling (purple) in trunk skeletal muscles in combination with dark-field imaging revealing the shape of the skin, myotomes, and notochord. (C) Quantification of proportion of the different phenotypes observed in DMSO-treated atrofish larvae (n = 33) or atrofish larvae after 24 (n = 36), 48 (n = 32), or 72 (n = 28) hours of 4-OHT treatment, with phalloidin incorporation and F310 immunostaining. Significance is determined by contingency Fisher’s exact test exact. (D) Confocal sections of DMSO-treated atrofish larvae (top) or atrofish larvae treated with 4-OHT for 24 hours (bottom) at 6 dpf showing phalloidin incorporation (green), A4.1025 (purple) and F310 (cyan) immunolabelling in trunk skeletal muscles. White arrow shows phalloidin incorporation in the absence of F310 expression but presence of A4.1025 expression. Asterisks show sites of muscle-fiber degeneration. Scale bars: 100 μm. (TIF) [file pgen.1012019.s003.tif]

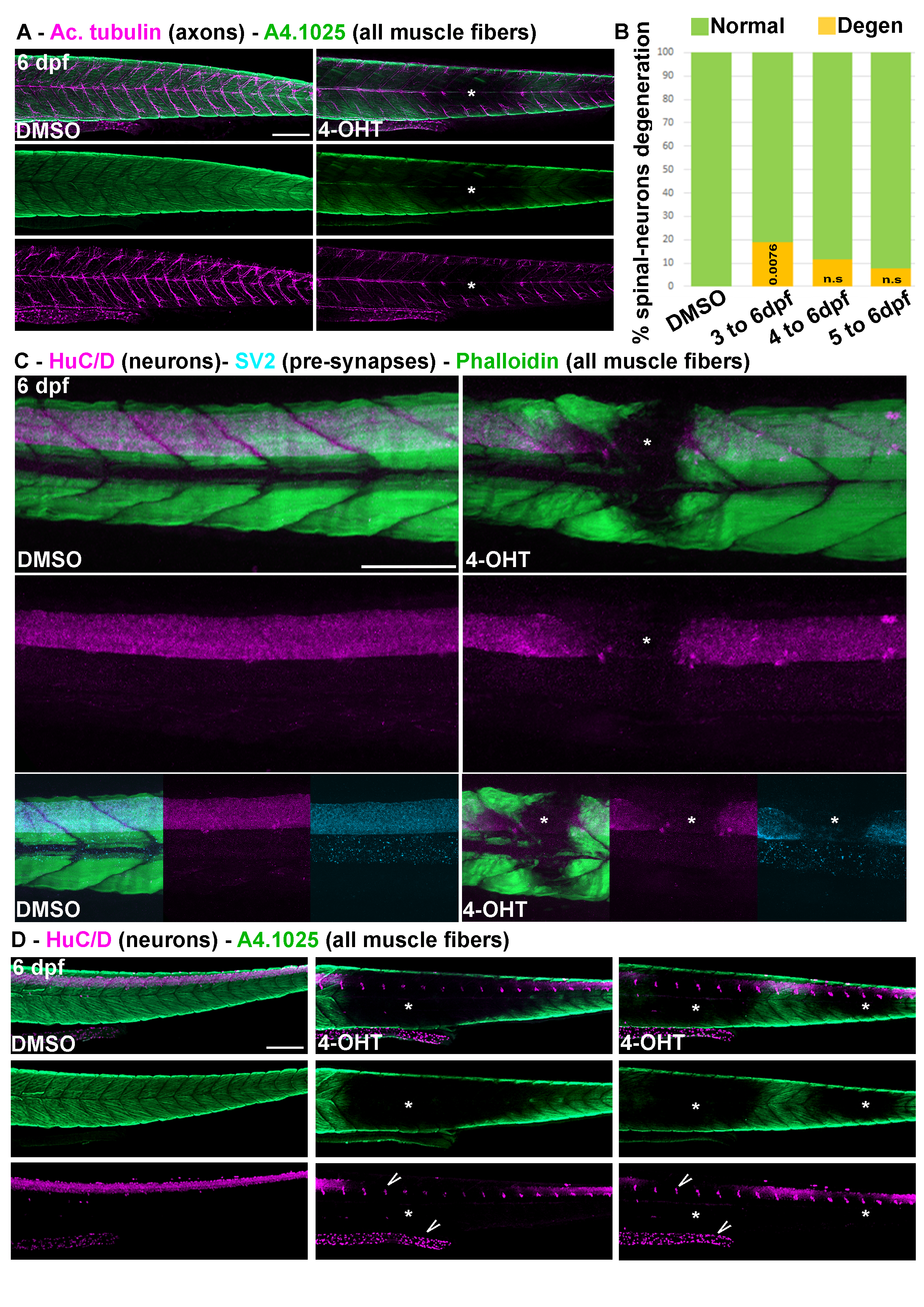

Supplement: S4 Fig — (A) Apotome sections of DMSO-treated atrofish larvae (left) or atrofish larvae treated with 4-OHT for 48 hours (right) at 6 dpf showing reduced acetylated tubulin (purple) and A4.1025 (green) immunolabelling in trunk skeletal muscles. Asterisks show sites of muscle-fiber degeneration. (B) Quantification of proportion of the different phenotypes observed in DMSO-treated atrofish larvae (n = 32) or atrofish larvae after 24 (n = 38), 48 (n = 35), or 72 (n = 32) hours of 4-OHT treatment, with HuC/D immunostaining showing spinal neurons. Significance is determined by contingency Fisher’s exact test exact. (C) Confocal sections of DMSO-treated atrofish larvae (left) or atrofish larvae treated with 4-OHT for 24 hours (right) at 6 dpf showing phalloidin incorporation (green), HuC/D (purple), and SV2 (cyan) immunolabelling in trunk skeletal muscles. Bottom row shows phalloidin, HuC/D, and SV2 staining in the central region of the trunk muscles shown in the top row. (D) Apotome sections of DMSO-treated atrofish larvae (left) or atrofish larvae treated with 4-OHT for 72 hours (middle and right) at 6 dpf showing reduced HuC/D (purple) and A4.1025 (green) immunolabelling in trunk skeletal muscles. Asterisks show sites of muscle-fiber and spinal-neuron degeneration. Dorsal root ganglia in the peripheral nervous system (top arrow) and neurons in the gut (bottom arrow) are not affected by muscle degeneration. Scale bars: 100 μm. (TIF) [file pgen.1012019.s004.tif]

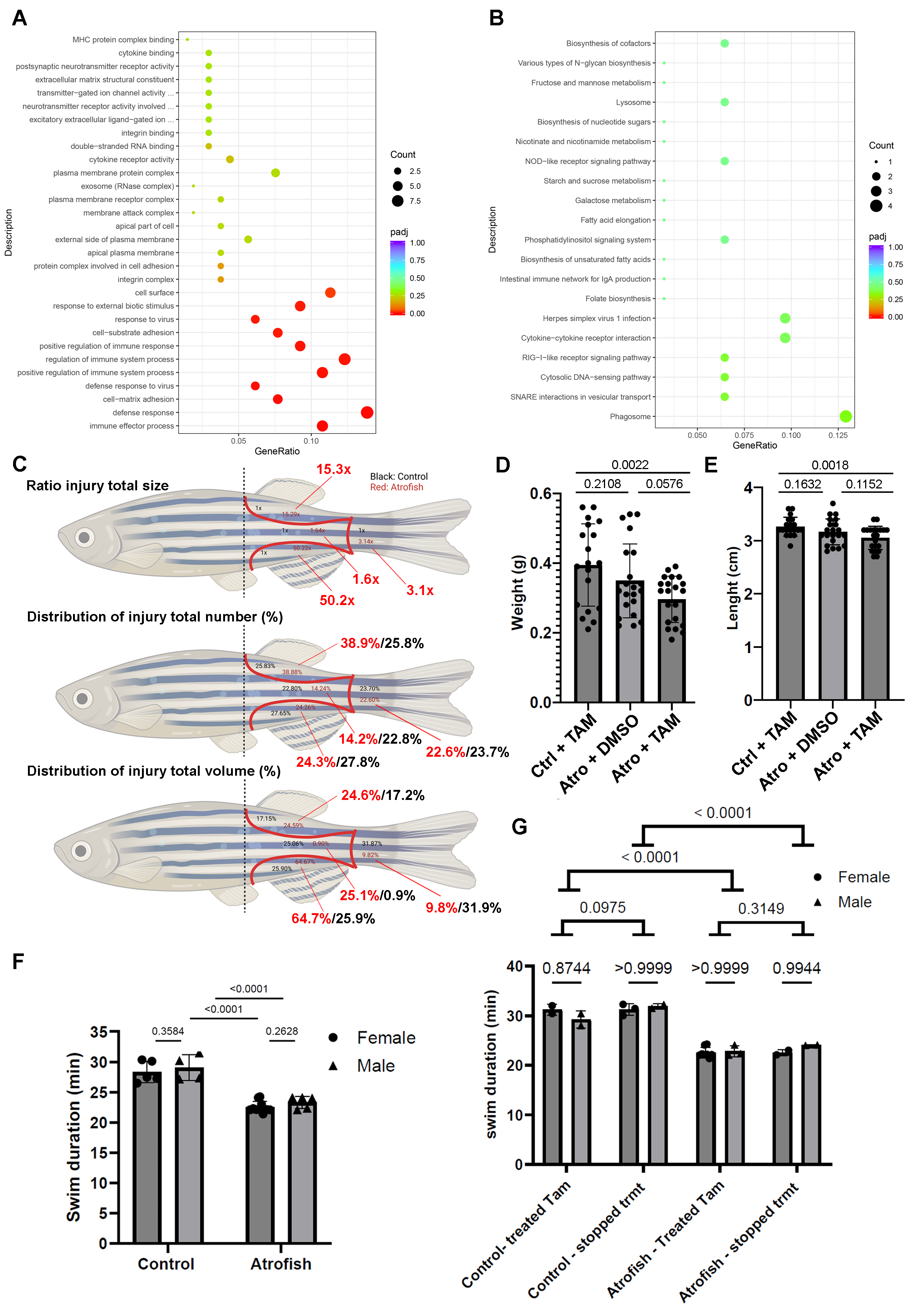

Supplement: S5 Fig — (A) GO (Gene Ontology) between atrofish larvae treated with DMSO for 24 hours and atrofish larvae treated with 4-OHT for 24 hours, with all larvae then placed in chemical-free medium for 48 hours until 8 dpf. (B) KEGG analysis between atrofish larvae treated with DMSO for 24 hours and atrofish larvae treated with 4-OHT for 24 hours, with all larvae then placed in chemical-free medium for 48 hours until 8 dpf. (C) Schematic representation of injury locations and distribution (distribution = number of injuries in a particular location as a percentage of the total number of injuries) in control fish (black) and atrofish (red) treated with TAM for 5 months until 6 months of age: ratio of injury size (top), number of injuries (middle), and volume of injuries (bottom) between control and atrofish. The red line separates the different muscle regions of interest for the analysis. (D) Quantification of fish weight in control fish (n = 19) treated with tamoxifen (TAM) or atrofish treated with either DMSO (n = 21) or TAM (n = 21). Statistical significance is determined by Mann-Whitney test, two-tailed, unpaired. (E) Quantification of fish length in TAM-treated control fish (n = 19) or atrofish treated with either DMSO (n = 21) or TAM (n = 21). Statistical significance is determined by Mann-Whitney test, two-tailed, unpaired. (F) Quantification of swimming capacity of control fish and atrofish at 6 months of age, treated with either DMSO (n = 9) or 4-OHT (n = 15) for 5 months, represented by gender. Statistical significance is determined by a Two-way Anova. (G) Quantification of swimming capacity of control fish and atrofish at 9 months of age, treated with TAM for 5 months until 6 months of age, and then treated for with either DMSO (n = 9) or TAM (n = 15) for an additional 3 months (until 9 months of age) to test for recovery of swimming function, represented by gender. Statistical significance is determined by a Two-way Anova. Error bars represent s.d. S5C in S5 Fig: Created [file pgen.1012019.s005.tif]
