# Supplementary material for: Zebrafish genetic model of neuromuscular degeneration associated with Atrogin-1 expression
Source: PLoS Genet. 2026 Jan 9;22(1):e1012019. doi: 10.1371/journal.pgen.1012019 (PMC12810921; doi:10.1371/journal.pgen.1012019)
Supplement: Attachment — Submitted filename: PloS Genet_Atrofish_Rebuttal_Final.docx [file pgen.1012019.s014.docx]

**Reviewers' comments:**

We would like to thank the reviewers for their supportive and constructive comments, and we apologize for the delay in resubmitting as we needed to establish new transgenic backgrounds for some of the suggested experiments. Please see the response below.

**Reviewer #1:** This study by Menard et al. aims to provide a new model to study sarcopenia in larval zebrafish. Key findings are: (1) the development of a new gain-of-function Atrogin-1 line, specifically in skeletal muscle, (2) loss of both muscle and neurons in the model, and (3) ability of zebrafish to recover from the atrophy. The fact that overexpression of Atrogin-1 causes atrophy is perhaps the most provocative result of this study as mutants of Atrogin-1 also have muscular atrophy. That said, much of the data is primarily descriptive with little mechanistic insight provided. Overall, the problem is important, the topic interesting, but experimental support for the claims is inadequate. Specific concerns are listed below:

We are extremely grateful for the reviewer’s supportive comments regarding our findings and the importance of our work for the field. We understand the reviewer’s concern about some experimental approaches (although we do not believe that our approaches are inadequate), and we discuss and address these points below. However, while some of our data are descriptive, as would be expected for the characterization of a new genetic tool, we would like to stress that in addition to other information on the atrofish phenotype, we showed that the following molecular mechanisms drive inflammation and underlie the atrophic phenotype: myosin light-chain degradation following Atrogin-1 expression, with motoneurons subsequently impacted by muscle degeneration and elevated cortisol levels.

1) The authors nicely show that treatment with 4-OHT causes mCherry expression in the muscle fibers and when crossed to the Atrogin-1 fish, a range of muscle atrophy is observed. However, there is no confirmation that Atrogin-1 is consistently upregulated after treatment. Given the range of phenotypes (which can happen with ablation studies but also with imperfect Cre recombination), finding a way to quantify the amount of Atrogin-1 that is overexpressed is critical to evaluate the rest of the paper. This is particularly important as tamoxifen is known to have an effect on muscle atrophy and fatty degeneration in mammalian models.

We agree with the reviewer’s point regarding Antrogin-1 expression, and we would like to clarify aspects of our experimental approach. In contrast to mCherry expression, we do not necessarily expect “Atrogin-1 to be consistently upregulated in muscle fibers after treatment,” as mentioned by the reviewer. Indeed, Atrogin-1 expression is predicted to induce muscle atrophy and degeneration, indicating that some muscle fibers will die after Atrogin-1 expression (and consequently will no longer express *atrogin-1*). In fact, we wanted to analyze Atrogin-1 expression at the protein level to distinguish between levels of exogenous *loxP-stop-loxP-atrogin1* mRNA that will allow for translation of exogenous Atrogin-1 protein when *Lox*P-site recombination takes place, and the levels required when *Lox*P recombination does not take place. Unfortunately, although we have tested many Atrogin-1 antibodies, including two that are published in fish, we have been unable to validate their efficiency and/or specificity. However, we demonstrated by RNAseq that *atrogin-1* mRNA levels can be increased in atrofish larvae versus control larvae (see below), indicating that endogenous Atrogin-1 expression is increased over fourfold (4.4) in atrofish after 24 hrs of 4-OHT treatment. This information has been added to the manuscript (p. 12).

In addition, we now provide an additional panel in Figure 1A revealing the spatial organization of *atrogin-1* expression using HCR RNA FISH, and showing an approximately ninefold increase in expression in trunk skeletal muscle tissue after 48 hours of treatment. Whether this increased expression shown by HCR FISH is exogenous and/or endogenous cannot be investigated in our current experimental setup and in the absence of working antibodies directed against the Atrogin-1 protein. We have now included a new panel (Fig. 1A; p. 9) in the manuscript.

Regarding the comment about tamoxifen having an impact on muscle atrophy, we again agree with the reviewer. We actually mentioned this effect on muscle homeostasis in the previous version of the manuscript (p. 21): “This effect could be attributed to a moderate positive impact of TAM treatment on muscle tissue, as studies in mice have suggested that TAM could have a beneficial effect on muscle hypertrophy and function[48–51].” Throughout the experiments described in the manuscript (in particular during the locomotion test), we used control larvae with 4-OHT or control adults with TAM to account for this effect.

2) It is not clear why overexpression of Atrogin-1 and knockout of Atrogin-1 would both cause muscular atrophy and increased apoptosis. The authors mention many times a feedback-loop component to this pathway. It would help the reader if a schematic of this feedback loop (known or proposed by the authors) was available.

While we understand that it may be puzzling that both gain of function and loss of function produce the same phenotype, this observation has no direct connection with the positive feedback loop mentioned in the manuscript. However, we agree that a schematic would help the reader, so we added a figure (Fig. 6; p. 22) to summarize our findings.

We would like to emphasize that overexpression of Atrogin-1 (E3 ubiquitin ligase) was first shown in models of fasting in mice, where its overexpression induced muscle atrophy (PMID: 11717410; PMID: 15109499; PMID: 11679633). Similarly, mouse genetic models of Atrogin-1 overexpression also exhibit muscle-atrophy phenotypes (PMID: 15109499). The literature involving Atrogin-1 overexpression in muscle atrophy and sarcopenia is extensive. In fact, a recent study of the zebrafish mutant for Atrogin-1 (PMID: 38530354) mentioned: “A unifying feature of these pathologies is the upregulation of members of the E3 ubiquitin ligase family, resulting in increased proteolytic degradation of target proteins.” We believe that our work is consistent with the previously published mechanism of increased expression of an E3 ubiquitin ligase, such as Atrogin-1, inducing muscle atrophy. We added this information about Atrogin-1 loss and gain of function in the introduction (p. 6-7), in particular regarding the central role of proteolysis in the etiology of muscle atrophy.

Further, have the authors tried using their overexpression line in the context of the published mutant? Can overexpression of Atrogin-1 rescue the initial atrophy and then additional time/increase concentration of 4-OHT show new development of atrophy? While it may be impractical to generate a double transgenic in the mutant line, a CRISPANT-based approach could get at this without the need to make multiple lines.

While we understand the rationale behind this experiment, we are not convinced that it will help clarify or support our work, regardless of the result. We believe that it is outside the scope of this study to try to rescue a mutant that we have not generated and are not using in our analysis. Based on the arguments presented above regarding the established effect of Atrogin-1 overexpression on muscle atrophy in mammals, we hope that the reviewer will understand our point of view.

3) Sarcopenia is primarily induced (although there are other factors) by changes in muscle synthesis signaling pathways. This leads to increased fat infiltration which can then drive inflammation and reduce strength and activity of the muscles. In Figure 1, the RNAseq suggests inflammation is occurring as cytokines are upregulated. However, there is no further analysis to confirm that the muscle atrophy is modeling sarcopenia (i.e. fat infiltration) and not a cytokine-mediated or inflammation-based atrophy like cachexia. This is critical given the accelerated timeline of the atrophy.

The reviewer is correct that sarcopenia is a complex and multifactorial disease, with fat infiltration being one of many symptoms associated with an atrophic phenotype in mammals. Atrophy mediated by inflammation is also a symptom/hallmark of sarcopenia in mammals.

Atrogin-1 overexpression is a main indicator of sarcopenia disease in mammals, as it controls proteolysis in muscle fibers (as noted above, this role is well established), which is the reason we chose to use this experimental strategy in fish. As suggested, we have now performed additional experiments regarding the fat-infiltration phenotype, using Nile red to assess whether or not Atrogin-1-dependent atrophy correlates with fat infiltration. Our data indicate that in zebrafish larvae, Atrogin-1 overexpression has no significant effect on fat infiltration (new Fig. S2B; p. 11-12). The quantity of fat deposition is low in control larvae (in the absence of a high-fat diet), as previously reported at this stage of development (PMID: 28348140; PMID: 36386866).

4) The neuronal staining is not compelling. For example, in Figure 2, the authors show that in an atrophied-muscle section, there is a loss of signal in the zn8 staining. But zn8 staining also labels commissural fibers, outer surface of somites, some ganglia and radial glial cells. In SF3, HuC/HuD staining shows a similar absence of staining. However, there is little explanation as to why the staining in the spinal cord is completely missing in just 24-48 hours. It takes time for neurons to die, particularly neurons that are not directly in contact with muscle fibers (sensory tract, interneurons, etc).

The reviewer is correct that it takes time for neurons to die. This is the likely explanation for our observation of only a small percentage of larvae with spinal neurons missing when the treatment is performed for only 24 hours (~5%, Fig. S4B). The percentage showed an increase with an increase in the time of 4-OHT treatment (72 hours, ~20%, Fig. S4B). As indicated in the Fig. S4D figure legend, the larvae that showed an absence of staining in a large portion of the spinal cord were treated for 72 hours.

Given so much of the staining in the spinal cord is gone – could the lack of signal just be due to increased fibrosis or fat infiltration which precludes the antibody from properly staining the spinal tract? This possibility seems even more likely given that HuC/HuD neurons are intact in the sensory bundles across the lateral line, which are also in contact with the atrophied muscle. The zebrafish community has a large number of plasmids and transgenic lines that label motor neurons, interneurons, and other spinal tract cells. Although getting the transgenic overexpression system into the lines may be challenging, plasmid injection would confirm loss of cells without the use of an antibody. Alternatively, staining for neuronal death with apoptosis markers or a using a plasmid-based approach (SecA5) in conjunction with the zn8 antibody at earlier timepoints would make the loss of signal more convincing.

We thank the reviewer for this comment and we agree with the suggestion. Accordingly, we have now performed additional experiments regarding the fat infiltration and fibrosis phenotype using Nile red and Sirius red respectively, and didn’t observe any modifications of these processes using these staining. We have also now performed experiments using the Tg(*mnx1*:mCherry) transgenic line labelling motoneurons. We showed that spinal motoneurons are also missing when we perform the analysis based on endogenous mCherry expression under the *mnx1* promoter (Fig. 3A, B, p. 16). We have also now quantified the neurodegenerative phenotype (Fig. 3B, C). In addition, using markers of fibroblasts/fibrosis (*pdgfrb*) or radial glial cells/gliosis (*gfap*), we observed no accumulation of these cells at sites of motoneuron degeneration in the spinal cord or in muscle tissue of atrofish larvae (Fig. S2B-E; p. 11-12).

5) The locomotor experiments are not consistent with the staining or each other. In Figure 3, Atrofish 4-OHT have no swimming at the time of treatment (0 hr). 4-OHT takes time to absorb into the larvae and then it takes time for Cre to cause recombination. Why are the transgenic fish already not swimming at the time of 4-OHT application? Are these fish orally gavage or injected with the 4-OHT? There needs to be a timeline of imaging (phallodin staining or the line, whichever is easier) that correlates loss of locomotion with muscle atrophy, and neuronal loss. Otherwise, what is the locomotion reporting on – muscle function or loss of neurons?

We apologize for the insufficient description of the timing of the treatment, which may have led to a misunderstanding. Our original text included: ”We first demonstrated that 4-OHT-treated atrofish larvae show reduced locomotion between 6 and 8 dpf compared to DMSO-treated atrofish larvae and 4-OHT-treated wild-type (WT) larvae (Fig. 4A).” And our original figure legend for Fig. 4A included: “Larvae were treated with DMSO or 4-OHT for 24 hours between 5 and 6 dpf before the locomotion test.” Actually, larvae were incubated in 4-OHT for 24 hours before the beginning of the locomotion test. We have clarified the timing of treatment on p. 18 of the manuscript (and additional movie S3), and we hope that this information alleviates the reviewer’s concern about “transgenic fish already not swimming” and the lack of correlation between locomotion and atrophic phenotypes.

6) Similarly, it doesn’t make sense that at the time of washout, the Atrofish 4-OHT swim comparably to DMSO fish. Once recombination with the Cre has occurred, it is permanently overexpressed. It would take the proliferation of cells that didn’t recombine to stop the overexpression of Atrogin-1. Other larval studies with SMA, ALS, and SCI models, show it takes >24 hr for motor neurons to regenerate in 6 dpf larvae. Even if no axons that project from the brain were damaged, without the proper CPG patterning, normal free swim would not be possible within the timeframe of 4-OHT metabolism. Do the larvae exhibit different movements as compensatory actions?

We understand the reviewer’s comment. We agree that once *Lox*P sites have recombined, cre would be expressed until death of the muscle fibers, and neuronal regeneration would take more than 24 hours. However, we disagree with the comment “it doesn’t make sense.” We were also not expecting to observe this rapid recovery, but we did indeed observe this in multiple experiments; “surprising” might better describe our finding. We now provide one additional movie of the locomotion recovery (Movie S6).

We would like to clarify that only a small percentage (~5%) of atrofish exhibit severe neuronal degeneration after 24 hours of 4-OHT exposure (Fig. S4B). This data may account for the rapid recovery of atrofish larvae (many of them swimming after 24 hours of recovery, Movie S6). In our manuscript we did not mention that the swimming recovery was driven by neuronal regeneration. Based on the timing of the recovery, we believe that it is not the case. We have now discussed and clarified the point about neuronal regeneration (p.18 in the manuscript).

**Reviewer #2:** This manuscript introduces and characterizes transgenic zebrafish lines (Atrofish) that conditionally overexpress the ubiquitin ligase Atrogin-1 in muscles. The authors show that this system effectively induces muscle degeneration in larvae and adults. Their characterization suggests that inflammation is induced by muscle degeneration in Atrofish larvae, myosin light chain is affected by Atrogin-1 overexpression, muscle degeneration causes non-autonomous neuronal defects in larvae, larvae can recover from Atrogei-1-induced injury, and Atrofish can be used to cause degenerative phenotypes in adults.

This model could be used to further investigate the function of Atrogin-1, and is valuable as a model for characterizing and developing treatments for muscle degeneration. The data presented are shown clearly and, for the most part, analyzed appropriately. Additional analyses, however, would strengthen the manuscript by providing a more thorough characterization, making the model more valuable. Suggestions for further analyses are described below. Not all should be necessary for publication, but each could clarify the phenotypes observed.

We are extremely grateful for the reviewer’s supportive comments regarding our study characterizing a new transgenic model of atrophy in zebrafish. We also greatly appreciate the suggestions to strengthen our work.

Major comments:
1. Transcriptional analyses suggests an increase in inflammation in Atrofish. This is an interesting observation, and suggests that the immune system may play a role in the Atrofish phenotype. Further insight into this idea could be provided by imaging innate immune cells (macrophages and neutrophils) in relation to muscle damage. This can be done in zebrafish relatively easily with stains or immunostaining, or even better, with live imaging of macrophage reporter lines that could be followed in live animals during degeneration.

We thank the reviewer for this comment. As suggested, we have now performed additional experiments regarding the inflammation phenotype using the *mpeg1:mCherry* transgenic line. Using this line, we did not observe an increase in the total number of macrophages (Fig. S2A; p.11). However, using HCR fluorescent *in situ* for the *mpeg1.2* gene in combination with the *mnx1:mCherry* transgenic line labeling motoneurons, our observations indicate that the localization of macrophages may differ compared to non-injured control larvae; i.e., we observe macrophages in degenerated muscle tissue, particularly in close vicinity to fragmented motoneuron axons and NMJ. These data are now included in Fig. 3C; p. 16.

2. By staining with F310, the authors show that loss of myosin light chain accompanies muscle atrophy, and suggest that loss of myosin light chain may precede overt disruption of muscle fibers, as imaged with Phalloidin. This conclusion is based on the observation that in a small number of cases (<10% with 1-day OHT exposures, according to Figure S2C) F310 staining is impaired without obvious loss of phalloidin staining. For this argument to be more convincing, the authors should perform a time-course, which, if there conclusion is correct, may demonstrate that F310 is lost earlier after exposure than phalloidin staining is lost. (The most convincing way to test the role of myosin light chain in degeneration, which may be beyond the scope of this report, would be to determine if overexpression of the light chain in Atrofish delays muscle degeneration).

We agree with the reviewer, and we apologize for the incomplete description of the timing of myosin light-chain degradation and phalloidin incorporation in the atrofish. As mentioned by the reviewer, this conclusion is based on Fig. S3C; however, other data of ours also support this conclusion. In particular, Fig. 2A, B show this phenomenon (see below for an example of strongly reduced myosin light-chain staining (magenta) in a myotome where phalloidin (green) is still incorporated). We agree that a longitudinal analysis of the same larvae would strengthen our study. Unfortunately, this experiment would require live imaging of myosin light-chain degradation, and we currently lack the genetic tools for this analysis. We have clarified the timing of myosin light-degradation (p. 14 in the manuscript) and we hope that this information alleviates the reviewer’s concern about this phenotype.

3. The observation that motor neurons are affected non-autonomously in Atrofish is interesting, but this observation could be presented more clearly and quantified better. For example, the defects in motor neurons are difficult to see in Figure 2C with the znp-1 and zn-8 staining. Higher magnification/higher resolution images could help, as well as tracing and quantification of axon length and synapse density. Using a transgenic line that labels motor neurons clearly would be a better way to characterize motor axons. Finally, a time course quantifying axon degeneration over time after Atrogen-1 induction, relative to muscle degeneration, would be most convincing.

We thank the reviewer for this comment. As suggested, we have now performed additional experiments regarding the neuronal phenotype using the Tg(*mnx1*:mCherry) transgenic line labelling motoneurons. We show that spinal motoneurons can also be affected when we perform the analysis based on mCherry expression under the activity of the *mnx1* promoter (Fig. 3A). In addition, using confocal imaging and 3D reconstruction for the analysis, we have now quantified the volume of cell bodies in the spinal cord, the global volume of motoneuron axons in the peripheral system, axon branching, and NMJ density in control and atrofish using the *mnx1*:mCherry reporter line (Fig. 3B). These data are now included in Fig. 3A, B, and on p. 16 of the manuscript.

4. The loss of staining for neurons throughout an entire segment of the spinal cord (Fig S3C) is surprising and could be exciting, but staining with HuC/D is not the best way to appreciate this phenotype. Loss of staining for this antigen may not be equivalent to loss of neurons. Staining for cell death and/or using a transgenic line to label all neurons would provide insight. It’s hard to imagine how all the neurons in a part of the spinal cord could die, but one possibility is that inflammation in that region is killing neurons and/or impeding staining. Imaging macrophages and microglia, as suggested in point 1 above, could address this possibility.

As indicated above, we have now performed an experiment using *mnx1:mCherry* labelling motoneurons to demonstrate that mCherry fluorescent cell bodies can be lost in the spinal cord (Fig. 3A). Finally, while imaging macrophages using the *mpeg:mCherry* line or HCR *in situ*, we did not observe increased accumulation of macrophages in the spinal cord in atrofish in comparison to control larvae.

5. The recovery experiments are surprising. Data previously presented in the manuscript shows that 5-OHT treatment, even for 24 hours, leads to cellular damage of muscles and neurons. Yet, if I understand the data correctly, immediately after removing fish from 5-OHT they behave completely normally (Figure 3B). This does not seem likely to be enough time to repair cellular damage (i.e., activate stem cells to make new muscle fibers, regrow MN axons and synapses). Transcriptional analyses are insufficient to characterize this “recovery” process. Staining for muscle and motorneurons at mulutiple timepoints is necessary to understand this process. The best way to do this experiment would be with a transgenic reporter that labels muscles, so that atrophy and recovery could be monitored longitudinally in live fish.

We understand the reviewer’s comments, and we agree that this result is surprising. We were also not expecting this rapid recovery, but we did indeed observe this result in multiple experiments. We would like to clarify that only a small percentage of atrofish exhibit severe neuronal degeneration after 24 hours of 4-OHT exposure (Fig. S4D). This data may account for the recovery of the atrofish larvae (many of them swim after 24 hours of recovery, Movie S6). As mentioned by the reviewer, the period of time following removal from 4-OHT treatment is likely too brief for stem-cell activation and regeneration. In our manuscript we did not indicate that the swimming recovery was driven by neuronal regeneration. Based on the short amount of time following removal from treatment, we believe that it is not the case. We have now discussed and clarified the point about neuronal regeneration (p.18 in the manuscript).

We agree that longitudinal analysis with live imaging would be the best experiment to perform. As suggested by the reviewer, we have now used transgenic animals expressing mCherry in motoneurons and GFP in muscles to perform this analysis. Unfortunately, when using 4-OHT treatment with larvae embedded in agarose with tricaine for anesthesia, we observe very limited degeneration, supporting our observations that muscle contraction is involved in development of the degenerative phenotype. Based on this observation, we were unable to perform live imaging of neuromuscular degeneration and regeneration. This information is now discussed in the manuscript (p. 22).

Minor comment:

1. For the analysis of muscle fibers presented in Figure 1B, the specific criteria distinguishing mild and strong phenotypes should be described.

Thank you for this comment. We have now clarified the wording regarding the different phenotypes.
