# Supplementary material for: Zebrafish genetic model of neuromuscular degeneration associated with Atrogin-1 expression
Source: PLoS Genet. 2026 Jan 9;22(1):e1012019. doi: 10.1371/journal.pgen.1012019 (PMC12810921; doi:10.1371/journal.pgen.1012019)
Supplement: Attachment — Submitted filename: PloS Genet_Atrofish_RebuttalV2RM.docx [file pgen.1012019.s015.docx]

**Reviewers' comments:**

Response to the Reviewers

**Reviewer #1:** The authors present a new genetic model for muscular atrophy using overexpression of atrogin-1 as a driver for accelerated muscular atrophy. The authors have added new live imaging studies which strengthen their conclusions. I am in favor of publication.

We are grateful for the reviewer’s supportive comments regarding our work,.

**Reviewer #2:** This revised manuscript describes a zebrafish model of Atrogen-mediated muscle degeneration. The authors have revised the manuscript with additional experiments, analyses, and text revisions. The authors have not been able to answer all reviewer comments, but they have carried out the most feasible suggestions and met the reviewer comments halfway.

Key revisions that have improved the manuscript include better quantification of Atrogin-1 expression level increases in Atrofish, staining for fat infiltration, using a motor neuron transgenic line to better appreciate motor neuron degeneration, and using a transgenic line and in situ hybridization to assess the role of macrophages in inflammation. In particular, the use of the motor neuron line has improved the characterization of the motor neuron degeneration phenotype.

Notwithstanding the improvements to the manuscript, a few concerns remain that the authors should address more directly, at least with additional discussion.

We appreciate the reviewer’s supportive comments regarding our additional experiments. We are also thankful that the reviewer understands the fact that live imaging is the only reliable experiment that can provide new insights into the myosin light-chain degradation phenotypes, and that this type of analysis will require the generation of new genetic tools as part of a future study. Regarding the recovery phenotype, we have now added a more developed discussion of the potential mechanisms underlying this process, and we have added a new panel (Figure 3A) regarding the control experiment and the effect of 4-OHT (or the absence of its negative effect) on neurons. The reviewer’s comments are discussed in more detail below.

1. The explanation for the quick recovery of swimming behavior after 4-OHT removal is unsatisfying. If it is not a regenerative response that repairs damage, then how would this work? Is it that the 4-OHT is toxic and somehow synergizes with degeneration in Atrofish?

The explanation for this result in the text is inadequate. Perhaps I’m missing something, but I do not understand what the authors mean when they write that “preventing atrogin-1 overexpression [presumably by removing 4-OHT] in muscle fibers allows for an efficient return to a homeostatic and functional state.” How would removing 4-OHT prevent overexpression? Presumably the Cre recombination has already taken place and overexpression is continuing.

Since this is a confusing section of the manuscript, the authors should discuss a plausible explanation for interpreting this “recovery” result. In the absence of a feasible interpretation, this finding is not meaningful and could be misleading--if that is the case, the authors should consider removing it from the manuscript.

We understand that the swimming-recovery phenotype is surprising, but we believe that it is important to retain this information in the manuscript, as it describes a component of the overall atrofish phenotype. As discussed in text we added to the first revision based on reviewer 1’s comment, removing 4-OHT would potentially prevent additional fibers from recombing *Lox*P sites and overexpressing atrogin-1. However, we understand the reviewer’s concerns, and we also agree that once *Lox*P sites are recombined, exogenous atrogin-1 will be expressed until the death of the muscle fibers. Finally, the endogenous atrogin1 expression that we identified to be upregulated in the atrofish may also be reversed by removing the 4-OHT, and participate to the recovery.

It is also important to note that following 24 hours of 4-OHT treatment, most (if not all) larvae exhibit defective swimming, but only a small percentage of atrofish (~5%) exhibit severe neuronal degeneration (Fig. S4B) and only ~25% exhibit mild or severe muscle degeneration (Fig. S1B). Together, these observations indicate that mechanisms other than atrogin-1 overexpression could underlie the defective locomotion. We have added this information in the Discussion section, as well as hypotheses regarding the quick-recovery phenotype (p. 18-19). These hypotheses are based on data in the paper regarding redundant mechanisms affecting locomotion. Specifically, we discuss the possible ole of inflammation, the stress response, and ECM remodeling in the defective locomotion behavior. For example, a change in ECM composition can affect muscle contraction, and this process, as well as inflammation and the stress response, could potentially be reversed more quickly than would be possible via the degree of cell regeneration required to recover normal swimming function. We hope that the reviewer will understand that we want to base our hypotheses regarding the quick recovery on data presented in the manuscript and not extrapolate outside of our findings. Our more thorough discussion also includes possible experiments for testing the role of inflammation and the stress response in the quick-recovery phenotype.

2. The evidence suggesting that loss of mlc precedes muscle degeneration remains relatively weak without a time course experiment. I appreciate that a longitudinal experiment is not feasible to do in live animals, but the authors could do a time-course by staining batches of animals at different time-points after induction to determine if there are early time points when there are more mlc-negative, intact muscles. Alternatively, since this isn’t a critical point in the paper, it would be acceptable for the authors to discuss this result more equivocally.

We agree with the reviewer that live imaging would enable the best interpretation of the mlc phenotype. However, this experiment would require the generation of new genetic tools to analyze mlc stability, and therefore would need to be part of a future project. As shown in Fig. S3C, we have tested different treatment time-points. In addition, as suggested by the reviewer, we have added a discussion of the timing of the treatment and the tools needed to better understand this phenotype (p. 15).

3. The authors sometimes appear to be using 4-OHT in wildtype animals as a control, sometimes use DMSO with Atrofish as a control, and sometimes use both controls. It is not clear why they chose to use different controls in different experiments, but the results are strongest when both controls are used. In particular, it would be reassuring to see the 4-OHT/WT control for the motor neuron/spinal cord degeneration experiments, rather than just the DMSO/Atrofish control. This would rule out the possibility that the hormone is detrimental to neurons.

The reviewer is correct that we used different controls in different experiments. We originally used DMSO with atrofish to control for a potential leak of ERT2creERT2 activity. Analyses of both fixed tissues and locomotion tests using living larvae strongly indicate that in the absence of 4-OHT, we observe no negative effects on muscle homeostasis or function, indicating that our genetic model using creERT2 can reliably be induced specifically by 4-OHT treatment.

As mentioned by the reviewer, we used additional controls with 4-OHT or TAM in control/WT fish when performing a swim test in larvae or adults, respectively. As we discussed in the manuscript, we chose this strategy because TAM has been shown to improve muscle function and to lead to muscle hypertrophy. We also observe a small improvement in swimming function when control fish are treated with TAM. This observation suggests that the treatment itself is not responsible for the reduced locomotion observed in atrofish after atrogin-1 expression. The information above has been added in the manuscript (p. 22).

The reviewer’s comment regarding the neuronal phenotype is fair, and we have now performed a new set of experiments to assess this effect. We demonstrate that that 4-OHT treatment in control (wt) mnx1:mcherry transgenic larvae has no negative effect on neuronal survival or the neuromuscular system (p.16). Supporting this observation, a previous publication indicates that TAM treatment in mice also has no significant negative effect on neurogenesis or behavioral response (PMID: 28203140). Instead, we observed a slight increase in motoneurons after 4-OHT treatment, possibly a scaling response as suggested by previous literature (PMID: 32516570). This information has also been added to the manuscript (p. 22). See below for representative images.

4. It is not clear how to reconcile the data in S2A showing no changes in macrophages in the transgenic line with the HCR data showing an increase in the macrophage transcript mpeg (Figure 3C). This discrepancy should be discussed.

The reviewer raises a good point. The discrepancy the reviewer refers to is likely the result of imprecision in our explanation of the data. We did not actually state that Fig. 3C showed an increase in macrophages; rather, we mentioned that the location of *mpeg*+ macrophages may differ in atrofish, i.e., they are accumulated in atrophic muscle tissue. We have now clarified our interpretation of the data (p. 16).

Also, it is important to note that the promotor used in the transgenic line and the probe for the HCR *in situ* experiment are reporters for different genes. The transgenic line was made using the *mpeg1.1* promoter while the HCR probe targeted the *mpeg1.2* gene (because it was more strongly upregulated in our RNAseq analysis). Our use of different genes may have contributed to differences in the results, as the two genes may respond differently at the transcriptional level and/or may not be expressed in the same subpopulations of immune cells. Ideally, we would have like to combine the *mnx1* and *mpeg1.1* transgenic lines. However, we had to use the alternative approach (HCR in situ) because it was not possible to combine the *mnx1* and *mpeg1.1* transgenic lines as they both express the mCherry fluorophore. In the manuscript, we have now clarified how the difference between the reporter line and the *in situ* probe may have affected on the results (p. 16).

5. The Zn8 staining in Figure 2 is very grainy and looks like much of it may be background staining. This staining is less clear than zn8 staining I’ve seen in other papers, suggesting that there may be a problem with the authors’ batch of antibody. Since the znp-1 staining and transgenically labeled motor neurons make the same point, this zn8 staining adds little to the manuscript and could probably be removed.

As suggested by the reviewer, we have now removed the zn8 immunostaining from Fig. 2C.

Minor point:

1. What are the yellow blobs in the 3D reconstructions shown in Figure 3B? They are not explained in the text or figure legend.

We apologize for the insufficient information in the legend for Fig. 3. The muscle tissue was imaged with *mnx1:mCherry* and HCR *in situ* for *mpeg1.2*. The yellow cells are *mpeg1.2*+ cells that were segmented and were therefore not quantified in the analysis. We have added a color code to the Fig. 3 legend indicating the colors corresponding to the different biological components of the tissue.
